# Stress-Associated Changes in MiR-20b-3p as a Potential Predictor of Underlying Psychopathology in the Depressed Brain: Mechanistic Insights from a Rat Model of Chronic Restraint Stress

**DOI:** 10.1007/s12035-025-05309-2

**Published:** 2025-11-15

**Authors:** Sarah Ali, Yogesh Dwivedi

**Affiliations:** Department of Psychiatry and Behavioral Neurobiology, University of Alabama at Birmingham, Birmingham, AL 35242 USA

**Keywords:** Stress, MiRNA, Brain, Animal model, MDD, HPA-axis, MRNA and miRNA expression

## Abstract

Major depressive disorder (MDD) is strongly linked to chronic stress, yet its molecular mechanisms remain poorly understood. We hypothesize that chronic stress induces epigenetic alterations in stress-associated genes, contributing to transcriptional dysregulation in the prefrontal cortex (PFC), a key brain region implicated in MDD. To test this, we performed expression levels of stress-associated genes in the PFC of rats subjected to restraint stress, which revealed significant downregulation of *CRHR2* and *NR3C1*, both critical regulators of the hypothalamic–pituitary–adrenal (HPA) axis, as well as a trend to decreased expression of *BDNF* and *TRKB*, central mediators of synaptic plasticity. Given the well-established role of miRNAs in gene regulation, we further investigated stress-associated miRNA expression changes and identified miR-20b-3p and miR-425-3p as significantly upregulated in the restraint-stressed group. Bioinformatics analysis predicted miR-20b-3p as a potential regulator of *NR3C1*, a relationship confirmed through RISC-mediated immunoenrichment, demonstrating increased miR-20b-3p binding to *NR3C1* in stress-exposed rats. To confirm this interaction, we performed parallel analyses in cells ectopically expressing miR-20b-3p mimic, inhibitor, and a non-oligo control. Cells mimicking endogenous miR-20b-3p showed upregulated *NR3C1* binding enrichment compared to non-transfected controls, while anti-miR-20b-3p treatment repressed its interaction with the *NR3C1* 3'UTR. These findings suggest that miR-20b-3p actively engages with *NR3C1* and plays a regulatory role in its expression. Collectively, these results highlight a potential epigenetic mechanism by which chronic stress alters *NR3C1* expression, possibly contributing to HPA axis dysregulation in MDD. The identification of miR-20b-3p as a key regulator of *NR3C1* provides new insights into stress-induced molecular changes and suggests potential therapeutic targets for stress-associated psychiatric disorders.

**Supplementary Information:**

The online version contains supplementary material available at 10.1007/s12035-025-05309-2.

## Introduction

Major depressive disorder (MDD) is a debilitating psychiatric illness with a lifetime prevalence of 20.6% [1]. In 2018, the World Health Organization (WHO) ranked MDD as the third leading contributor to the global burden of disease, with its increasing prevalence continuing to worsen its negative impact [2, 3]. MDD encompasses a range of physical and psychological symptoms, including anhedonia, sleep disturbances, appetite changes, feelings of worthlessness, and difficulty concentrating [2, 4]. Given the severity and complexity of MDD, it is also important to recognize the elevated risk of suicide, with recent reports suggesting that approximately 60% of individuals who die by suicide have MDD as their primary psychiatric diagnosis [5]. The heterogeneity of MDD poses challenges in diagnosis and management. Approximately 60% of depressed patients experience recurrent episodes, with each successive episode carrying a 10–20% risk of incomplete remission [6]. Increasing knowledge regarding the pathology of the MDD brain has underscored the need to explore the underlying molecular circuitry. Many of these circuits acquire maladaptive changes that contribute to the development and progression of MDD. However, because of their extreme complexity, many of the intricate molecular mechanisms in the MDD brain remain poorly understood and need further empirical study support.

One key factor that contributes to MDD and its chronic nature is the dysregulation of the hypothalamic–pituitary–adrenal (HPA) axis, a central part of the body’s response to stress. This neuroendocrine link helps to maintain homeostasis by regulating the release of glucocorticoids (GCs) like cortisol in humans and corticosterone (CORT) in rodents, which are crucial for adaptive stress responses. While acute stressors may produce protective effects (allostasis), repeated sympathetic activation from persistent chronic stress can become maladaptive (allostatic load). Under normal circumstances, stressors trigger the release of corticotropin-releasing factor (CRF) from the hypothalamus, leading to the secretion of adrenocorticotropic hormone (ACTH). This, in turn, triggers the synthesis and release of GCs, which bind to glucocorticoid receptors (GRs) in limbic and prelimbic brain regions, including the prefrontal cortex (PFC), where they play a crucial role in modulating the stress response. However, under chronic stress, this negative-feedback system is impaired, leading to maladaptive physiological as well as morphological changes, including elevated blood plasma cortisol concentrations, altered dendritic spine morphometry, and synaptic reorganization [7–9]. The stimulated HPA axis can also induce other persisting changes in the PFC area, like atrophy [10]. These changes are decisive in preventing the PFC from performing its emotional and cognitive roles. Previous studies have corroborated these findings in maladaptive neuroplasticity and have shown to contribute to the failure of the PFC to respond to environmental challenges [11, 12].

Emerging evidence suggests that epigenetic modifications, such as microRNA (miRNA)-mediated gene regulation, may be a key mechanism underlying the pathological effects of chronic stress and the development of MDD. miRNAs are small non-coding RNAs that regulate gene expression through post-transcriptional RNA silencing. Dysregulation of miRNA expression has been linked to alterations in the expression of target genes that regulate the stress response, neuroplasticity, and HPA axis function, making miRNAs a promising area of study for understanding the molecular mechanisms of MDD [13, 14]. Previously, our lab identified transcriptome-wide miRNA alterations in the PFC of rats following chronic CORT treatment [15] and demonstrated the role of miRNAs in mediating stress resilience and susceptibility to depressive symptomatology [16, 17]. Building on these findings, other studies from our group in MDD subjects showed unique miRNA expression patterns, with specific miRNAs regulating GR in the PFC and affecting synaptic plasticity [18, 19].

To further elucidate the role of miRNAs in shaping stress-responsive pathways implicated in depression pathophysiology, we explored the molecular consequences of chronic stress exposure within the cellular environment of the PFC, focusing on miRNA-driven regulation of transcripts involved in neuroendocrine signaling and synaptic plasticity. Utilizing a well-established animal model of chronic restraint stress, we assessed expression changes of key HPA axis and plasticity-associated genes, alongside a cohort of miRNAs with established relevance to stress. It is pertinent to mention that restraint-stress rats have been extensively characterized in prior studies to exhibit behavioral changes resembling depression-like phenotypes [20–22]. Through a literature search, we identified 10 miRNAs—miR-124-3p, miR-504, miR-137-5p, miR-132-5p, miR-20b-3p, miR-135-3p, miR-34a-5p, miR-425-3p, miR-29a-3p, and miR-34c-5p—that have been previously associated with stress-induced gene expression changes in various preclinical and clinical studies [18, 23–26]. We further utilized a target prediction algorithm from the TargetScan database, focusing on cumulative weighted context +  + (CWC + +) scores for the presence of conserved 8mer, 7mer, and 6mer sites matching the seed region of each miRNA. Our analysis revealed significant downregulation of *NR3C1* and *CRHR2*, accompanied by upregulation of miR-20b-3p and miR-425-3p. Among these, miR-20b-3p emerged with a compelling regulatory relationship to the GR gene, *NR3C1*, supported by both in vivo and in vitro evidence consistent with a direct post-transcriptional interaction. Collectively, these findings position miR-20b-3p as a critical molecular effector of stress-induced *NR3C1* suppression, pointing to an epigenetic mechanism by which chronic stress compromises neuroendocrine stability. This interaction not only enhances our understanding of HPA axis vulnerability in MDD but also highlights miR-20b-3p's potential as a target for therapeutic intervention and opens avenues for examining its translational relevance in human depression.

## Materials and Methods

All experiments adhered to the National Institutes of Health (NIH) guidelines for the care and use of laboratory animals and received approval from the University of Alabama at Birmingham's Institutional Animal Care and Use Committee (IACUC).

### Animals

Adult male Sprague–Dawley rats (250–300 g) were obtained from Envigo (Indianapolis, IN, USA) and housed in pairs under standard laboratory conditions (21 ± 1 ºC, 55 ± 5% humidity, 12-h light/dark cycle). One week prior to the start of experiments, the rats were provided ad libitum access to food and water and allowed to acclimate to the laboratory environment. They were then randomly assigned to either the handled-control group or the chronic restraint group.

### Chronic Restraint Stress

Restraint stress was administered during the light cycle (08:00–12:00). Each rat was placed in a clear acrylic tube (20 cm length, 6.35 cm inner diameter) with air vents along the tube and cap, allowing the tail to extend outward from the rear end. The cap was secured at a depth that restricted mobility within the tube. Rats underwent restraint for 2 h daily over 14 consecutive days. Handled-control rats were exposed to the restraint apparatus daily but were not subjected to restraint. For this study, 6 handled-control rats and 6 restraint-stressed rats were used for mRNA and miRNA assays. For in vivo immunoenrichment investigations, 5 handled-control rats and 5 restraint-stressed rats were used. Our sample size justification is supported by prior work [27], which identified 57 differentially expressed miRNAs in the hippocampal dentate gyrus of stressed rats, with several (e.g., miR-204-5p, miR-1298, miR-143-3p, miR-34c-5p) showing log2 fold changes of ≥ 1 and highly significant differences (*p* < 0.0001) using only 7–8 animals per group. Such findings indicate large effect sizes. Consistent with this, our power analysis (α = 0.05, 80% power) shows that 5–6 animals per group are sufficient to detect similarly large effects (Cohen’s d ≈ 1.6–2.0), thereby validating the adequacy of our proposed sample size.

### Tissue Collection

Twenty-four hours following the final restraint session, each animal was anesthetized with isoflurane, and blood was drawn via thoracotomy and cardiac puncture into EDTA tubes. The whole blood was centrifuged at 1400 rpm for 15 min at 4 °C to separate the plasma as indicated [17]. The rat brains were extracted and flash frozen in liquid nitrogen. The PFC region was carefully dissected with a scalpel using the *Rat Brain Atlas* as a reference. All tissue samples were then stored at −80 °C for further analysis.

### Plasma-Based CORT Enzyme-Linked Immunosorbent Assay and PFC RNA Isolation

CORT levels in rat plasma, retrieved at the time of sacrifice, were quantified using an enzyme-linked immunosorbent assay following the manufacturer’s instructions (Enzo Life Sciences, ADI-901–097).

Total RNA was extracted from PFC tissue using TRIzol reagent (Life Technologies, USA) as outlined earlier [28]. Chloroform was added to facilitate phase separation, after which the RNA-containing aqueous phase was collected. Isopropanol was used to precipitate the RNA, and the mixture was incubated overnight at –30 °C. Glycogen was used to optimize the procedure. The resulting RNA pellet was washed with 70% ethanol to remove contaminants, briefly air-dried, and resuspended in nuclease-free water. RNA purity was evaluated using a NanoDrop spectrophotometer (Thermo Fisher Scientific, Waltham, USA), ensuring a 260/280 absorbance ratio of ≥ 1.8. Additionally, RNA integrity was assessed via agarose gel electrophoresis. All samples included in the study had an RNA integrity number > 8.

### mRNA and miRNA First Strand cDNA Synthesis

A total of 500 ng of RNA isolated from the rat PFC was used to generate mRNA-specific complementary DNA (cDNA) with the ProtoScript First Strand cDNA Synthesis Kit (New England Biolabs, MA, USA). The oligo d(T) priming method was utilized to ensure cDNA synthesis initiated at the 3’ end of the transcript, while M-MuLV Reverse Transcriptase was incorporated to facilitate the reverse transcription process. Initially, the RNA was mixed with the oligo d(T)_23_ VN primer, denatured at 65 °C for five minutes, and then rapidly chilled on ice. Next, a 2X M-MuLV Enzyme Mix containing M-MuLV Reverse Transcriptase and murine RNase Inhibitor, along with a 10X MuLV Reaction Mix comprising dNTPs and an optimized buffer, was added. The 20 µl reaction was incubated at 42 °C for one hour, followed by enzyme inactivation at 80 °C for five minutes.

For miRNA cDNA synthesis, 500 ng of RNA was processed using a poly(A) tailing method with E. coli Poly(A) Polymerase enzyme (New England Biolabs, MA, USA). First, the RNA was combined with 1X E. coli Poly(A) Polymerase Reaction Buffer, E. coli Poly(A) Polymerase enzyme, 40 U RNase inhibitor, and 1 mM ATP, then incubated at 37 °C for 30 min. After the poly(A) tailing step, 1 µM oligo d(T) adapter primer was added, and the reaction was incubated at 60 °C for an additional 5 min, followed by a quick chill on ice. Lastly, reverse transcription was carried out by adding 2X M-MuLV Reaction Mix and 10X M-MuLV Enzyme Mix, incubating the mixture at 42 °C for 1 h, and finally, inactivating the enzyme at 80 °C for 5 min.

### Rat-Specific mRNA and miRNA Expression Primer Designing

Rat-specific mRNA gene primers were designed using the NCBI RefSeq database, while miRNA-specific primers were based on sequences from miRBase. To maximize efficacy, primers were positioned near the 3' end of the coding transcript. For individual miRNA amplification, only the forward primer was designed to complement a universal adapter-specific reverse primer sequence. Specificity for both gene and miRNA primers was ensured by conducting a homology search using the NCBI nucleotide (NT) BLAST tool, minimizing the risk of off-target amplification. The mRNA and miRNA primers used for expression analysis are detailed in Table 1.

**Table 1:** mRNA primer sequences for qPCR-based expression analysis

Primer Name	Sequence
rno_*GAPDH*_F	CAC TGA GCA TCT CCC TCA CAA
rno_*GAPDH*_R	TGG TAT TCG AGA GAA GGG AGG
rno_*FKBP5*_F	TGG CTG TAG TAA GTC GGT CA
rno_*FKBP5*_R	CAA CTC CGG GAA ACA AGT GA
rno_*CRHBP*_F	TCC AGC AAG TGA GGA AGA GAG C
rno_*CRHBP*_R	CGG TGA GTG CTG CCA TTT CTT
rno_*CRH*_F	CAA GCT CAC AGC AAC AGG AA
rno_*CRH*_R	ATT TTG TCC TAG CCA CCC CT
rno_*CRHR1*_F	ATG TTC GTC TGC ATT GGC TG
rno_*CRHR1*_R	TGC CAA ACC AGC ACT TTT CA
rno_*CRHR2*_F	GGA TGA CAA GCA GAG GAA GT
rno_*CRHR2*_R	AGC ACT AGG AAA AGC AGG AA
rno_*NR3C1*_F	CAG GGT CGG CTT CTG TCT A
rno_*NR3C1*_R	GGA GGG TAT TTT CAT ACA GCC A
rno_*BDNF*_F	TCT TGC TGT GGT CTC TTT TTG G
rno_*BDNF*_R	GAG AAC AAG GCC ACA GAC ATT T
rno_*TRKB*_F	GAC CTC CCA GCT ATT CGA GC
rno_*TRKB*_R	GAG TTG CCT CCC TAT CGC TG
Universal Reverse	GCG AGC ACA GAA TTA ATA CGA C
U6_F	CTC GCT TCG GCA GCA CA
U6_R	AAC GCT TCA CGA ATT TGC GT
rno_miR-124-3p	TAA GGC ACG CGG TGA ATG CC
rno_miR-504	CCT GGT CTG CAC TCT GAA AA
rno_miR-137-5p	CGG GTA TTC TTG GGT GGA TAA
rno_miR-132-5p	CGT GGC TTT CGA TTG TTA CT
rno_miR-20b-3p	GCA GTG TGA GCA CTT CTG
rno_miR-135-3p	GCT ATG GCT TTT TAT TCC TAT GTG AAA
rno_miR-34a-5p	GCA GTG TCT TAG CTG GTT G
rno_miR-425-3p	GAA TAT CGT GTC CGC CAA AA
rno_miR-29a-3p	TAG CAC CAT CTG AAA TCG GA
rno_miR-34c-5p	GCA GTG TAG TTA GCT GAT TGC A
rno_*NR3C1* 3’UTR_F	GCA CCG ATT GGT CTA GCT CT
rno_*NR3C1* 3’UTR_R	TGG CAC ATG TAG GGA TGT GT
rno_miR-20b-3p Seed Sequence	CTG CAG T

### qPCR-based mRNA and miRNA Expression Assay and Analyses

Relative mRNA and miRNA transcript abundance was quantified using the AriaMx real-time qPCR system (Stratagene, Santa Clara, USA) with expression primers mentioned in Table 1. Both mRNAs and miRNAs were amplified using 1X Luna Universal qPCR mix (New England Biolabs, MA, USA). Gene-specific forward and reverse primers were applied at a final concentration of 0.25 μM. Raw cDNA was diluted 40-fold for mRNA samples and ten-fold for miRNA samples. For mRNA amplification, the thermal cycling conditions started with an initial denaturation at 95 °C for 60 s, followed by 40 cycles of denaturation at 95 °C for 15 s and annealing at 60 °C for 30 s. For miRNA amplification, the initial and subsequent thermal conditions were the same, except that primer annealing occurred at 55 °C for 30 s. Analysis of non-template samples facilitated the optimization of miRNA primers, ensuring the prevention of dimer formation and off-target product amplification. Relative mRNA transcript levels were normalized to *Gapdh*, and U6 was used for miRNA expression normalization. The relative differences in mRNA and miRNA transcript amplification were calculated using Livak’s ΔΔCT method [29], providing a quantitative measure of expression variation between the groups.

### In VitroCell Line-Based miRNA-Oligo Transfection Assay

Synthetic double-stranded nucleotide oligos (Dharmacon GE Life Sciences, Lafayette, USA) mimicking endogenous miR-20b-3p (cat # C-320489–03–0005) and its inhibitor (cat # IH-320489–04–0005) were transfected into rat pheochromocytoma PC-12 Adh cells (ATCC cat # CRL-1721.1). A control group was included in which cells were treated with the transfection reagent alone, devoid of any nucleotide oligos. The cells were cultured in Kaighn's Modification of Ham's F-12 Medium (F-12 K) supplemented with 2.5% fetal bovine serum, 15% horse serum, and 10,000 U/mL penicillin–streptomycin. The cells were maintained at 37 °C in a 5% CO₂ incubator, with the medium replenished every 24–48 h. Transfection of all miRNA oligos was carried out using Lipofectamine RNAiMAX (Invitrogen, Grand Island, USA) following the manufacturer’s instructions, and cells were harvested 48 h post-transfection. The transfected cellular lysates were stored in RIP (RNA immunoprecipitation) lysis buffer (150 mM NaCl, 50 mM Tris–Cl pH 7.4, 1 mM EDTA, 1 mM DTT, 0.5% NP-40, 1 × Halt Protease Inhibitor Cocktail, 200 U/ml RNaseOUT) for RISC-IP-based RNA immunoenrichment analysis.

### Ago2 Antibody-Mediated RNA-Induced Silencing Complex Immunoprecipitation (RISC-IP) Assay in Rat Brain and In-Vitro Cellular Lysates

To determine the physiological interaction between miRNA and its target mRNA transcripts, an antibody-based immunoenrichment (RISC-IP) procedure was performed following the method described in our previous publication [18]. Initially, rat PFC (15 mg) was homogenized in RIP lysis buffer, with 10% of the clear lysate set aside as an input control. Prewashed protein A/G magnetic beads (Thermo Fisher Scientific, Waltham, USA; cat # PI88803) were conjugated with recombinant Argonaute-2 (Ago2, cat # C34C6) rabbit monoclonal antibody (Cell Signaling Technology, cat # 2897S) and incubated overnight with the clarified tissue and cellular lysates. Afterward, the RNA was isolated from RISC using TRIzol reagent to capture bound miRNAs and their target mRNAs. Following the same methodology, lysates were prepared from the cellular harvests obtained in oligo transfection experiments and used in the RISC-IP assay. RNA was then extracted from both the immunoprecipitated (IP) samples and the 10% input fraction following the same method as described in the rat brain experiment above. Following that, the first strand cDNA was synthesized using a miRNA oligo seed sequence (Table 1). The cDNA was then amplified by qPCR with primers (Table 1) targeting the mRNA sequence near the miRNA-binding site in the 3′ untranslated region (UTR), enabling the analysis of mRNA–miRNA interactions. To control variability in sample input and IP efficiency, qPCR data from IP samples were normalized to their corresponding input values.

### Statistical Analysis

All data were processed using SPSS software (V.25; IBM, IL, USA) for statistical analysis and are presented as mean ± standard error of means (SEM). Student’s *t*-test was used to compare mean expression levels between groups, including mRNA and miRNA changes in rat brain tissue, target gene expression in miRNA oligo-transfected cell groups (non-transfected control, mimic, and anti-miRNA), and RIP analysis of both in vivo and in vitro conditions. To determine significant differences between group means, we also conducted an independent samples t-test for expression and miRNA-mRNA enrichment. Statistical significance was assessed at a 95% confidence interval (*p* ≤ 0.05). The relative transcript levels of *Gapdh* and U6, used as normalizers, were consistent across groups. When assessing the variance within calculated ratios, one handled-control and restraint rat sample was excluded from mRNA analyses due to exhibiting high variance.

## Results

### Expression of Stress-Responsive Genes in the Rat PFC

**Fig. 1:**
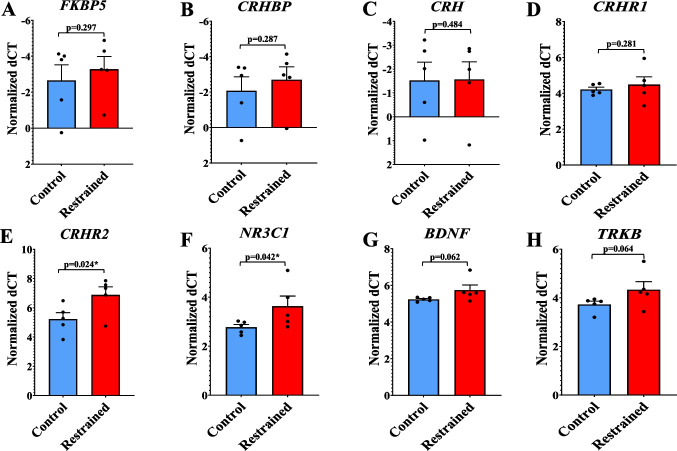
Stress-related mRNA transcript levels in the PFC of handled-control and restraint rats. Gene expression changes were calculated using the ΔΔCT from Student’s t-test and assessed using an independent samples *t*-test performed in SPSS statistical software. Data are displayed as bar diagrams with individual data points, where each dot represents an individual animal, with the group mean ± SEM indicated. Fold change expression is indicated in Supplementary Fig. 1.** A** The expression of *FKBP5* was elevated in the restraint-stressed rats, but this change was not statistically significant (*p* = 0.297, *F* = 0.876, *t* = 0.554, *df* = 8). **B** Relative transcript levels of *CRHBP* were upregulated for animals exposed to restraint stress; however, this increase did not surpass the threshold for statistical significance (*p* = 0.287, *F* = 0.233, *t* = 0.585, *df* = 8). **C ***CRH* gene expression showed no substantial differences between restraint-stressed and handled-control animals (*p* = 0.484, *F* = 0.142, *t* = 0.041, *df* = 8). **D ***CRHR1* expression was lower in restraint animals compared to controls, though this difference was not statistically significant (*p* = 0.281, *F* = 2.379, *t* = −0.605, *df* = 8). **E** A significant decline in *CRHR2* transcript abundance was observed for restraint-stressed rats (*p* = 0.024, *F* = 0.099, *t* = −2.330, df = 8). **F ***NR3C1* transcript expression was significantly reduced in restraint-stressed rats relative to control animals (*p* = 0.042, *F* = 6.264, *t* = −1.976, *df* = 8). **G ***BDNF* mRNA expression trended toward downregulation in restraint-stressed rats, but was not statistically significant (*p* = 0.062, *F* = 3.616, *t* = −1.724, df = 8). **H ***TRKB* expression followed a similar downward trend in restraint-stressed rats, and was not statistically significant (*p* = 0.064, *F* = 1.107, *t *= −1.700, *df* = 8). All mRNA expression data was normalized against *Gapdh*. *p ≤ 0.05

To assess the impact of chronic stress, gene expression analysis was performed in the PFC of handled-control and restraint-stressed rats. Stress-related gene targets relevant to depression pathophysiology were identified through a literature search and our previous studies [15, 18, 30–34]. The following mRNA transcripts were selected for expression analysis: *FKBP5*, *CRHBP*, *CRH*, *CRHR1*, *CRHR2*, *NR3C1*, *BDNF*, and *TRKB*. Gene expression changes are represented as fold change in Supplementary Fig. 1A-1H, while individual sample values are depicted as bar diagrams with individual data points in Fig. 1A-1H. For the gene expression data, a 2 (group) × 8 (mRNA) repeated measures ANOVA with multivariate tests showed significant mean differences across mRNAs (*p* = 0.0014) and a marginally significant group-by-mRNA interaction (*p* = 0.0656). Relative expression changes determined higher mRNA levels in restraint rats, with *FKBP5* increasing by approximately 54% (*p* = 0.297, *F* = 0.876, *t* = 0.554, *df* = 8; Figure S1A) and *CRHBP* by 55% (*p* = 0.287, *F* = 0.233, *t* = 0.585, *df* = 8; Figure S1B). These changes, however, did not reach statistical significance in an independent-samples t-test. Conversely, the restraint group exhibited decreased relative transcript expression across multiple genes, including *CRHR1*, which was lower by ~ 17% (*p* = 0.281, *F* = 2.379, *t* = −0.605, *df* = 8; Figure S1D), *CRHR2* (~ 68%; *p* = 0.024, *F* = 0.099, *t* = −2.330, *df* = 8; Figure S1E), *NR3C1* (~ 44%; *p* = 0.042, *F* = 6.264, *t* = −1.976, *df* = 8; Figure S1F), *BDNF* (~ 29%; *p* = 0.062, *F* = 3.616, *t* = −1.724, *df* = 8; Figure S1G), and *TRKB* (~ 34%; *p* = 0.064, *F* = 1.107, *t* = −1.700, *df* = 8; Figure S1H). Notably, both *NR3C1* and *CRHR2* showed statistical significance, whereas *TRKB* and *BDNF* showed a trend toward significance but fell short of the threshold in an independent-samples t-test. For the remaining target gene, *CRH*, no significant change was observed between the two groups, as the relative fold change showed negligible variation around 1.0 (*p* = 0.484, *F* = 0.142, *t* = 0.041, *df* = 8; Figure S1C).

### Plasma CORT Levels and Correlation with Stress-Responsiveness Gene Expression

**Fig. 2:**
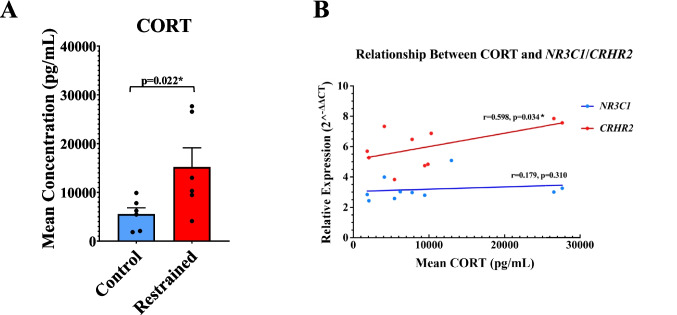
Plasma CORT concentrations and correlation with significant stress-responsive genes. The differences in CORT levels, shown as mean concentration (pg/mL), were determined in 5 handled-control and 5 restraint-stressed rats. Data are presented as mean ± SEM. **A** Plasma CORT concentrations were significantly elevated in the restraint group (*p* = 0.022), indicating stress-induced HPA axis activation. **B** Pearson correlation analysis was conducted to assess the relationship between circulating CORT levels and relative expression of two significantly downregulated genes in the rat PFC, *NR3C1* (blue) and *CRHR2* (red). *CRHR2* expression showed a significant negative correlation with CORT (*r* = 0.598, *p* = 0.034), while *NR3C1* exhibited a weaker, non-significant correlation (*r* = 0.179, *p* = 0.310). Each data point represents an individual animal. *p ≤ 0.05

To evaluate the physiological effects of restraint stress, plasma CORT levels were measured as an indicator of HPA axis activation. Rats subjected to restraint stress exhibited a significant increase in mean CORT concentration (15,172.997 pg/mL) compared to handled-control rats (5,540.943 pg/mL, *p* = 0.022, *F* = 8.799, *t *= −2.315, *df* = 10; Fig. 2A). This elevation reflected a sustained hormonal shift under chronic stress and established a physiological foundation for investigating downstream alterations associated with HPA axis dysregulation. Considering the significant downregulation of *NR3C1* and *CRHR2*, we explored whether their expression levels were associated with circulating CORT levels. Pearson correlation analysis revealed a significantly negative relationship between plasma CORT and *CRHR2* expression (*r* = 0.598, *p* = 0.034), while the correlation with *NR3C1* was not statistically significant (*r* = 0.179, *p* = 0.310; Fig. 2B). These results suggest that *CRHR2* expression may more directly reflect systemic GC activity, whereas additional mechanisms may regulate NR3C1 expression. We also correlated plasma CORT level with additional genes that did not show significant differences. Our results showed that none of these genes were significantly correlated with CORT (*FKBP*: *r* = 0.38, *p* = 0.13; *CRH*: *r* = 0.38, *p* = 0.14; *BDNF*: *r* = 0.12, *p* = 0.36; *TRKB*: *r* = 0.39, *p* = 0.12; *CRHBP*: *r* = 0.48, *p* = 0.08; *CRHR1*: *r* = 0.16, *p* = 0.32).

### Expression Profiling of Stress-Associated miRNAs in the Rat PFC

**Fig. 3:**
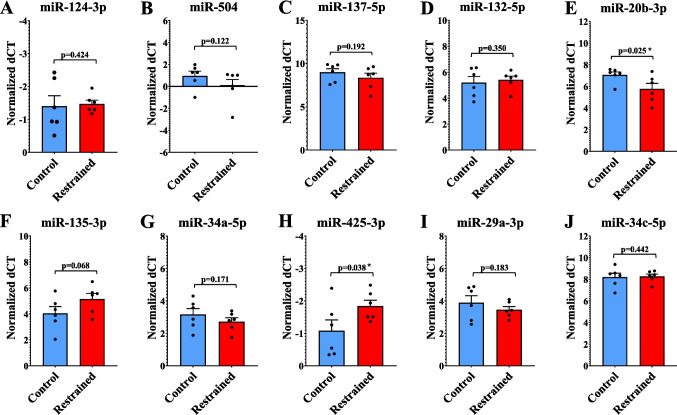
Differential expression of stress-related miRNAs in the PFC of handled-control and restraint-stressed rats. Data are displayed as bar diagrams with individual data points, where each dot represents an individual animal, with the group mean ± SEM indicated. Fold change expression is indicated in Supplementary Fig. 2. The relative miRNA transcript levels were analyzed using independent samples t-test in SPSS statistical software. Data are presented as mean ± SEM. 6 control rats and 6 restraint rats were analyzed. **A** For miR-124-3p, no substantial change was observed between restraint animals and handled-control rats (*p* = 0.424, *F* = 6.399, *t* = 0.198, *df* = 10). **B** miR-504 showed increased expression in the restraint group, though this change was not statistically significant (*p* = 0.122, *F* = 0.236, *t* = 1.240, *df* = 10). **C** Similarly, miR-137-5p expression was elevated in restraint-stressed rats but remained above the significance threshold (*p* = 0.192, *F* = 0.347, *t* = 0.909, *df* = 10). **D** miR-132-5p displayed a slight decrease in the restraint group without statistical significance (*p* = 0.350, *F* = 3.594, *t* = −0.396, *df* = 10). **E** In contrast, miR-20b-3p was significantly upregulated in restraint animals compared to controls, indicating a robust increase in expression in response to chronic stress (*p* = 0.025, *F* = 4.022, *t* = 2.219, *df* = 10). **F** miR-135-3p was downregulated in restraint-stressed rats, with a trend toward significance that did not meet the *p* ≤ 0.05 threshold (*p* = 0.068, *F* = 0.027, *t* = 1.620, *df* = 10). **G** miR-34a-5p exhibited an increase in the restraint group, but this change was not significant (*p* = 0.171, *F* = 1.953, *t* = 0.998, *df* = 10). **H** At a statistically significant level, miR-425-3p was elevated in restraint animals compared to controls (*p* = 0.038, *F* = 2.861, *t* = 1.984, *df* = 10). **I** miR-29a-3p expression increased following restraint stress, but did not reach significance (*p* = 0.183, *F* = 6.600, *t* = 0.948, *df* = 10). **J** miR-34c-5p expression remained stable across both groups, with no notable differences observed (*p* = 0.442, *F* = 1.288, *t* = −0.149, *df* = 10). All miRNA expression data was normalized against U6. *p ≤ 0.05

As we mentioned previously, stress-induced molecular changes in the brain are known to be driven by epigenetic mechanisms such as miRNA-driven gene regulation. To examine whether miRNAs contribute to mRNA expression changes in stress-associated genes in restraint-stressed rats, we conducted targeted expression analysis of candidate miRNAs. Through a literature search, we identified 10 miRNAs—miR-124-3p, miR-504, miR-137-5p, miR-132-5p, miR-20b-3p, miR-135-3p, miR-34a-5p, miR-425-3p, miR-29a-3p, and miR-34c-5p—that have been previously associated with stress-induced gene expression changes in various preclinical and clinical studies [18, 23–26]. Expression changes are represented as fold change (Supplementary Fig. 2A-J), while individual sample values are depicted as bar diagrams with individual data points (Fig. 3A-J). Our qPCR-based expression profiling of PFC from control and restraint-stressed rats revealed significant differences in a subset of miRNAs. In contrast, others showed directional changes that did not reach statistical significance. A standard repeated-measures ANOVA was performed to assess the overall significance of miRNA changes. We found significant mean differences across miRNAs (*p* < 0.0001) and a statistically significant group-by-miRNA interaction (*p* = 0.0007). Further analysis revealed that miRNAs miR-132-5p (*p* = 0.350, *F* = 3.594, *t* = −0.396, *df* = 10; Figure S2D) and −135-3p (*p* = 0.068, *F* = 0.027, *t* = −1.620, *df* = 10; Figure S2F) exhibited ~ 0.86- and 0.47-fold expression repression, respectively. Though the reduction in miR-135-3p approached statistical significance, it did not meet the *p* ≤ 0.05 cutoff. As some miRNAs showed reduced expression, others demonstrated increases in fold expression, including miR-504 (~ 1.91-fold, *p* = 0.122, *F* = 0.236, *t* = 1.240, *df* = 10; Figure S2B), −137-5p (~ 1.54-fold, *p* = 0.192, *F* = 0.347, *t* = 0.909, *df* = 10; Figure S2C), 34a-5p (~ 1.35-fold, *p* = 0.171, *F* = 1.953, *t* = 0.998, df = 10; Figure S2G), and 29a-3p (~ 1.36-fold, *p* = 0.183, *F* = 6.600, *t* = 0.948, df = 10; Figure S2I). These changes, however, were not statistically significant. Additionally, no significant changes were observed for miRNAs miR-34c-5p (~ 4% reduction, *p* = 0.442, *F* = 1.288, *t* = −0.149, *df* = 10; Figure S2J) or −124-3p (~ 0.05% elevation, *p* = 0.424, *F* = 6.399, *t* = 0.198, *df* = 10; Figure S2A). The remaining two miRNAs, miR-20b-3p (2.45-fold, *p* = 0.025, *F* = 4.022, *t* = 2.219, *df* = 10; Figure S2E) and miR-425-3p (1.69-fold, *p* = 0.038, *F* = 2.861, *t* = 1.984, *df* = 10; Figure S2H) showed transcript levels that were significantly higher in the restraint-stress group compared to the handled-control group.

### Identification of miRNAs Potentially Targeting Stress-Genes Following Prediction Analysis

Next, we investigated whether alterations in two of the most prominent genes (*NR3C1* and *CRHR2*) identified in our restraint-stress rat model could be attributed to miRNA-mediated changes. To do this, we utilized a target prediction algorithm from the TargetScan database, focusing on cumulative weighted context +  + (CWC + +) scores for the presence of conserved 8mer, 7mer, and 6mer sites matching the seed region of each miRNA. After conducting this analysis, we identified miR-20b-3p as the only miRNA with a strong potential to target *NR3C1, with a* CWC +  + score of −0.02.

### RISC-Mediated Immunoenrichment Assay to Identify Endogenous Binding of miR-20b-3p to NR3C1 in Rat PFC and miR-Oligo Transfected Cellular Model

**Fig. 4:**
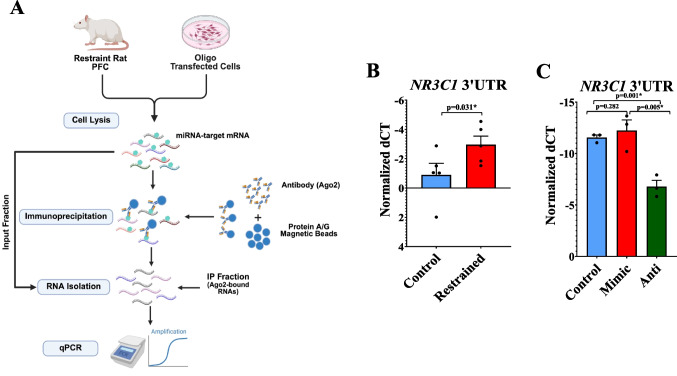
RISC-mediated immunoenrichment of *NR3C1* 3′UTR by miR-20b-3p in restraint-stressed rat PFC and miR-20b-3p oligo-transfected cells.** A** Schematic representation of the RIP workflow used to detect interactions between miR-20b-3p and *NR3C1* mRNA. PFC tissue from restraint-stressed rats, along with PC-12 Adh cells transfected with miR-20b-3p oligos, were lysed and subjected to Ago2-mediated RIP using Protein A/G magnetic beads and an Ago2 antibody. RNA was extracted from both input and IP fractions, and *NR3C1* 3′UTR enrichment was quantified by qPCR. Relative immunoenrichment was calculated by normalizing to input RNA. Data are displayed as bar diagrams with individual data points, where each dot represents an individual animal, with the group mean ± SEM indicated. Fold change expression is indicated in Supplementary Fig. 3. **B** In vivo analysis of PFC tissue from restraint-stressed (*n* = 5) and handled-control rats (*n* = 5) revealed a significant increase in *NR3C1* 3′UTR enrichment in the restraint group (*p* = 0.035, *F* = 0.029, *t* = 2.086, *df* = 8), suggesting enhanced miR-20b-3p-mediated targeting of *NR3C1* transcripts in response to chronic stress. **C** In vitro, PC-12 Adh cells were transfected with a miR-20b-3p agomir (mimic, *n* = 3) or inhibitor (anti-miRNA, *n* = 3), and compared to a non-transfected control group (*n* = 3). Although the increase did not reach statistical significance (*p* = 0.282), ectopic overexpression of miR-20b-3p was associated with enhanced binding to the *NR3C1* 3′UTR. In contrast, this interaction was markedly reduced in cells transfected with the miR-20b-3p inhibitor (*p* = 0.001), supporting the role of miR-20b-3p as a direct post-transcriptional regulator of *NR3C1*. All statistical analyses were performed using Student’s t-test and independent samples t-test in SPSS statistical software. Data are presented as mean ± SEM. *p ≤ 0.05. Ago2 = Argonaute 2; RIP = RNA immunoprecipitation; 3’UTR = 3’ untranslated region

To validate molecular changes within the brain, we further explored whether miR-20b-3p, as an epigenetic modifier, has any regulatory influence over *NR3C1* expression in restraint compared to handled-control rats. Fold change is represented in Supplementary Fig. 3A-B, while individual sample values are depicted as bar diagrams with individual data points in Fig. 4B-C. The RISC-mediated RNA immunoprecipitation (RIP) workflow used to assess this interaction is illustrated in Fig. 4A. Following qPCR testing, a highly significant four-fold binding enrichment of miR-20b-3p to the *NR3C1* 3’UTR was found for restraint rats (*p* = 0.035, *F* = 0.029, *t* = 2.086, *df* = 8; Figure S3A).

To determine whether this regulatory interaction persists in vitro, we performed parallel analyses in cells ectopically expressing miR-20b-3p mimic (*n* = 3), inhibitor (*n* = 3), or a non-oligo control (*n* = 3). The binding enrichment for miR-20b-3p on the *NR3C1* transcript in cells endogenously mimicking its expression showed ~ 1.60-fold expression compared to the non-transfected group, though not significantly (*p* = 0.282, Figure S3B). Notably, one replicate exhibited an extreme value; when this outlier is excluded, the enrichment became statistically significant (*p* = 0.02), supporting the expected interaction between miR-20b-3p and *NR3C1*. For cell groups treated with anti-miR-20b-3p, no binding enrichment was captured for the 3’ UTR of *NR3C1* (0.04-fold repression), and this result did reach statistical significance (*p* = 0.001, Figure S3B).

## Discussion

Chronic exposure to stressors is a key contributory factor in vulnerability to MDD, driving maladaptive alterations in stress-related neuroendocrine circuitry and impacting genes associated with GC signaling and neuroplasticity. The accumulation of these changes can trigger miRNA-mediated mechanisms that further modulate stress- and mood-related gene expression, ultimately impairing cognitive and affective processes. The present study provides evidence that chronic stress induces molecular reprogramming at both the mRNA and miRNA levels, linking HPA axis dysregulation to depression-like phenotypes in rodents.

To confirm the physiological impact of restraint stress, we first measured plasma CORT levels, which were significantly elevated in restraint-stressed rats. This suggests that chronic stress shifts HPA axis activity into a hyperactive state, a hallmark of neuroendocrine dysfunction widely observed in MDD [35, 36]. Notably, the significant increase in CORT corresponded with transcriptional changes in the rat PFC, where expression analysis revealed significant downregulation of two key stress-regulatory genes, *NR3C1* and *CRHR2*. Here, we argued that suppression of *NR3C1*, a key molecular mediator of stress hormone feedback that encodes the GR, likely disrupts the negative feedback loop responsible for limiting hypothalamic CRF and pituitary ACTH release, perpetuating CORT overproduction and prolonged HPA axis activation. Concurrently, reduced *CRHR2* may further exacerbate this dysregulation, as the receptor is integral to dampening CRF signaling and facilitating stress response. When *CRHR2* is diminished, the system’s ability to disengage from prolonged GC output is blunted, extending HPA axis activation beyond adaptive limits. Our correlation analysis further revealed that while *CRHR2* expression remained positively associated with circulating CORT, *NR3C1* exhibited no such relationship, suggesting potential divergence in their regulatory mechanisms under chronic stress. Such a pattern may reflect two outcomes. First, a decoupling between hormone levels and gene expression in the case of *NR3C1*, where elevated GCs no longer elicit the expected transcriptional response. Second, residual *CRHR2* responsiveness may reflect preserved, but altered, sensitivity to GC signaling that is insufficient to counterbalance the functional effects mediated by GR. This compromised shutdown mechanism compounds the feedback disruption driven by *NR3C1* loss, fueling a persistent neuroendocrine imbalance that mirrors the abnormalities consistently reported in patients diagnosed with MDD. Clinical studies have repeatedly demonstrated that individuals with MDD often exhibit hypercortisolism, as evidenced by elevated plasma and saliva cortisol concentrations, reflecting an overactive stress axis [37, 38]. Importantly, emerging evidence suggests that this hypercortisolemia can be associated with transcriptional and receptor-level alterations reminiscent of those observed in our animal model, strengthening the translational bridge between chronic stress exposure and the neurobiological substrates that underlie depressive illness [32, 39–41].

**Fig. 5:**
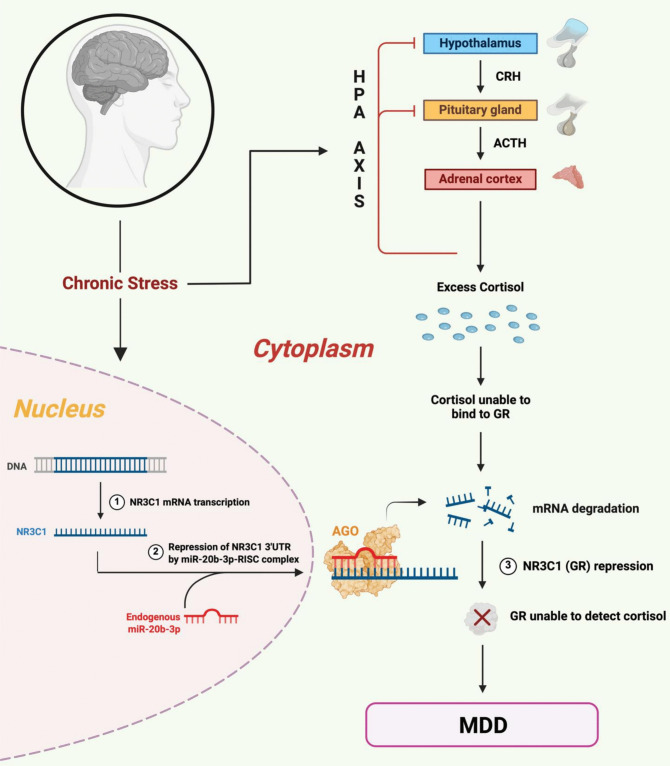
Hypothetical model depicting miR-20b-3p-mediated *NR3C1* suppression and HPA axis dysregulation under chronic stress. Chronic stress can induce the expression of miR-20b-3p, which enhances post-transcriptional silencing of *NR3C1* via RISC complex recruitment. This targeted repression reduces GR availability, impairing cortisol sensing and altering overall negative feedback regulation of the HPA axis. The resulting GR deficiency and sustained cortisol elevations drive neuroendocrine imbalance and may contribute to the onset and progression of MDD symptomatology. GR = Glucocorticoid Receptor

To uncover the mechanistic link underlying the repression of *NR3C1* and *CRHR2*, we next examined post-transcriptional regulation by stress-responsive miRNAs. From our selected panel of miRNAs, identified based on prior evidence implicating their roles in chronic stress adaptation and MDD-related molecular alterations, qPCR revealed a significant increase in miR-20b-3p and miR-425-3p in the PFC of restraint rats. Considering the established role of miRNAs in post-transcriptional gene silencing, we focused on miR-20b-3p because it showed responsiveness to stress exposure in the PFC of our rat model and was predicted to target NR3C1, as revealed by in-silico analysis. Our subsequent in vivo 3’UTR RISC-immunoenrichment assay confirmed direct binding of the miR-20b-3p seed sequence to *NR3C1*, suggesting that this miRNA may actively mediate the observed suppression of *NR3C1* under chronic stress conditions. Supporting this, our in vitro cellular model demonstrated enhanced binding to the 3’UTR of *NR3C1* in PC-12 cells ectopically overexpressing miR-20b-3p, which was significantly diminished upon miR-20b-3p inhibition. This mechanistic evidence also helps clarify the earlier correlation findings, as miR-20b-3p-mediated post-transcriptional repression may account for the decoupling of *NR3C1* from CORT levels, despite transcriptional inducibility, unlike *CRHR2*, which remained responsive. Our proposed hypothetical model, conceptualizing this interaction shown in Fig. 5, illustrates how chronic stress may lead to NR3C1 transcriptional repression and promote miR-20b-3p-mediated silencing, resulting in decreased GR availability, impaired cortisol sensing, and disruption of the HPA axis's negative feedback. These data collectively support the role of miR-20b-3p in sustaining cortisol elevations and promoting the emergence of MDD-related symptoms.

While acute upregulation of miR-20b-3p may initially represent a compensatory response aimed at tempering excessive GC signaling, sustained elevation likely impairs feedback regulation, promoting chronic stress vulnerability. This biphasic role aligns with prior studies showing that miRNA dynamics may transition from adaptive to maladaptive depending on the duration and intensity of stress exposure [19, 26, 42–44]. Intriguingly, we also observed a marginally significant decrease of *BDNF* and its receptor, *TRKB*, in restraint-stressed rats, while expression of miR-20b-3p was upregulated. This co-occurrence raises the possibility that miRNA dysregulation may not only mediate components of the HPA axis but also directly influence plasticity-related signaling cascades. Indeed, *BDNF* itself has been shown to modulate the GC pathway and homeostatic maintenance [45–47]. These changes also reflect molecular signatures often reported in the MDD brain, where decreased BDNF and TRKB expression have been linked to the development of MDD [48, 49]. The resulting reduction in neurotrophic support leads to neuronal atrophy, synaptic loss, and impaired neuroplasticity within regions highly involved in HPA axis activity [50]. Prior studies suggest that miRNAs may regulate these neuroplasticity-associated genes, highlighting their emerging role as epigenetic modulators of long-term stress adaptation [51, 52]. In this context, miR-20b-3p may serve as a key mediator of the brain's transcriptional response to chronic stress, shifting the balance between adaptive plasticity and pathological remodeling. This highlights a broader implication: chronic stress may disrupt endogenous miRNA signaling, causing lasting changes in neuroendocrine and neuroplasticity networks that, over time, may appear as core features of MDD. Moreover, fluctuations in miRNA expression may underlie individual differences in stress resilience versus vulnerability, with maladaptive miRNA responses heightening susceptibility to depressive symptoms. At the same time, more adaptive profiles may protect against stress-induced neuropathology. Together, these insights reinforce the potential of miRNAs as a convergent mechanism linking chronic stress exposure to the emergence and persistence of MDD.

One limitation of the present study is the use of only male animals, which limits the generalizability of findings. Because MDD exerts more pronounced effects in females, sex-biased transcriptomic differences, particularly involving components of the HPA axis, have been shown to be especially significant [53]. Similar sex-dependent patterns may also occur at the level of miRNA regulation, as supported by recent findings from our group [16, 17, 23]. Although the biological mechanisms underlying this sexual dimorphism remain unclear, gonadal hormones have been identified as potential regulators of mRNA and miRNA stress-linked neurocircuitry [54, 55]. This raises the possibility that sex-based hormonal variation could influence miR-20b-3p-mediated regulation of stress-responsive and neuroplasticity-related genes as well. Another important consideration is that our analyses were confined to the PFC but did not assess other interconnected limbic structures, such as the hippocampus and amygdala. These regions, alongside the PFC, form a core triad involved in mood regulation and HPA axis feedback [56, 57]. Interestingly, miRNAs have been shown to exert region-specific actions within this network. For example, miR-124 acts as a resilience factor in the hippocampus but promotes stress susceptibility in the PFC [58, 59]. It will be essential to investigate whether miR-20b-3p shows differential expression patterns and regulatory functions across this circuit. Such analyses will provide critical insight into how miRNAs fine-tune stress adaptation and contribute to vulnerability or resilience to depressive pathology, potentially in a sex- and region-dependent manner. Equally important will be the need to establish whether miR-20b-3p/*NR3C1* regulation translates into measurable effects on depressive-like behaviors. Future studies could address this by experimentally manipulating miR-20b-3p levels (e.g., overexpression or inhibition) in rodent stress paradigms and subsequently assessing anhedonia, despair, and HPA-axis feedback. Such approaches will be critical for establishing a causal role for miR-20b-3p in shaping stress-related behavioral outcomes and confirming its relevance to depressive pathology. Additionally, we observed a significant upregulation of miR-425-3p in the PFC of restraint-stressed animals. Although this miRNA was not prioritized for further analysis due to the absence of predicted interactions with our downregulated gene targets, miR-425-3p has been implicated in antidepressant effects [60]. In the future, we plan to characterize the gene networks and signaling pathways regulated by miR-425-3p better to understand the molecular basis of its antidepressant-like properties and evaluate its potential role in the development of depressive phenotypes within stress-related models.

Future research examining miRNA profiles in individuals with MDD holds promise for identifying predictive biomarkers that could aid in the early detection of stress-related disorders and help pinpoint individuals at heightened risk for developing chronic depression. It is increasingly recognized that miRNA networks, rather than single miRNAs, likely coordinate the regulation of essential stress- and plasticity-related genes [52, 61, 62]. Understanding how these miRNAs interact and collectively affect the stress axis is crucial to unraveling the complexity of HPA dysfunction in MDD. This understanding could be improved through more comprehensive animal models and human tissue studies, which would help confirm the role of miR-20b-3p and other stress-related miRNAs. Chronic stress is also known to activate inflammatory and oxidative stress pathways, which not only disrupt HPA axis regulation in depression, but also alter cellular stress responses [63, 64]. While miR-20b-3p has been minimally explored in the context of depression, its documented involvement in cancer, pulmonary fibrosis, and diabetes suggests a role in influencing cell proliferation, apoptosis, and autophagy [65–67]. These same cellular processes have also been implicated in the neurobiological alterations associated with chronic stress-induced MDD [64, 68]. In our rat model, we demonstrated that miR-20b-3p responds to stress and controls NR3C1 expression, providing initial evidence for a mechanistic link between chronic stress and HPA axis dysregulation. We acknowledge that *NR3C1* protein levels were not assessed. As miRNAs primarily act post-transcriptionally, evaluating GR protein expression will be essential to confirm that miR-20b-3p-mediated mRNA changes translate into functional alterations in HPA axis signaling. Due to the positioning and availability of tissue in this study, protein analyses could not be performed; but we plan to include these assessments in future experiments. We also note that the upstream mechanisms driving miR-20b-3p regulation by stress were not addressed in this study. Potential regulatory processes, such as GR binding at the miRNA promoter or miRNA promoter methylation, may contribute and represent important avenues for future investigation.

Altogether, our findings highlight the broader significance of miRNAs in orchestrating transcriptional responses to stress and driving neuroendocrine and neuroplastic changes associated with depression. Ongoing investigation into miRNA-mediated control of stress-related pathways will be critical for deepening our understanding of MDD and uncovering novel molecular targets for therapeutic development.

## Supplementary Information

Below is the link to the electronic supplementary material.

Supplementary file 1 (DOCX 406 KB)

## Data Availability

All data generated or analyzed during this study are included in this published article.
