# Supplementary material for: Stress-Associated Changes in MiR-20b-3p as a Potential Predictor of Underlying Psychopathology in the Depressed Brain: Mechanistic Insights from a Rat Model of Chronic Restraint Stress
Source: Mol Neurobiol. 2025 Nov 15;63(1):55. doi: 10.1007/s12035-025-05309-2 (PMC12619829; doi:10.1007/s12035-025-05309-2)
Supplement: Supplementary file 1 — (DOCX 406 KB) [file 12035_2025_5309_MOESM1_ESM.docx]

**Supplementary Section**

**Stress-Associated Changes in miR-20b-3p as a Potential Predictor of Underlying Psychopathology in the Depressed Brain: Mechanistic Insights from a Rat Model of Chronic Restraint Stress**

Sarah Ali, Yogesh Dwivedi^*^

Department of Psychiatry and Behavioral Neurobiology

University of Alabama at Birmingham

Birmingham, Alabama, USA 35242


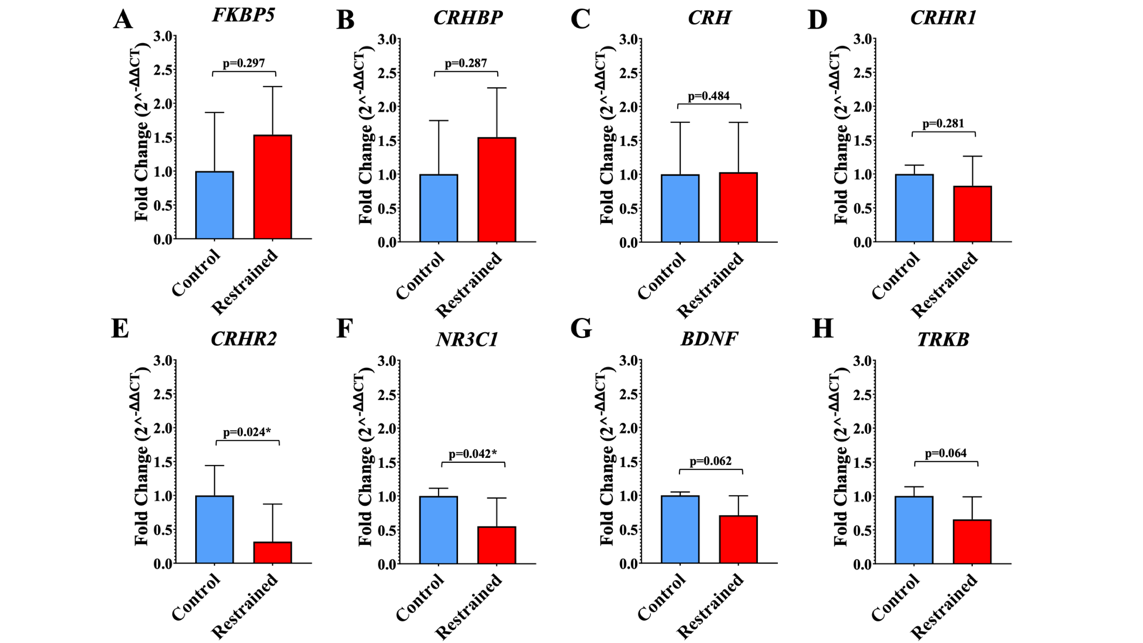


**Figure 1: Stress-related mRNA fold change in the PFC of handled-control and restraint rats**. Gene expression changes were calculated using the ΔΔCT from Student’s t-test and assessed using an independent samples t-test performed in SPSS statistical software. 5 handled-control rats and 5 restraint rats were analyzed. **(A)** The expression of *FKBP5* was elevated in the restraint-stressed rats, but this change was not statistically significant (p=0.297, F=0.876, t=0.554, df=8). **(B)** Relative transcript levels of *CRHBP* were upregulated for animals exposed to restraint stress; however, this increase did not surpass the threshold for statistical significance (p=0.287, F=0.233, t=0.585, df=8). **(C)** *CRH* gene expression showed no substantial differences between restraint-stressed and handled-control animals (p=0.484, F=0.142, t=0.041, df=8). **(D)** *CRHR1* expression was lower in restraint animals compared to controls, though this difference was not statistically significant (p=0.281, F=2.379, t=-0.605, df=8). **€** A significant decline in *CRHR2* transcript abundance was observed for restraint-stressed rats (p=0.024, F=0.099, t=-2.330, df=8). **(F)** *NR3C1* transcript expression was significantly reduced in restraint-stressed rats relative to control animals (p=0.042, F=6.264, t=-1.976, df=8). **(G)** *BDNF* mRNA expression trended toward downregulation in restraint-stressed rats, but was not statistically significant (p=0.062, F=3.616, t=-1.724, df=8). **(H)** *TRKB* expression followed a similar downward trend in restraint-stressed rats, and was not statistically significant (p=0.064, F=1.107, t=-1.700, df=8). All mRNA expression data was normalized against *Gapdh*. *p ≤ 0.05.


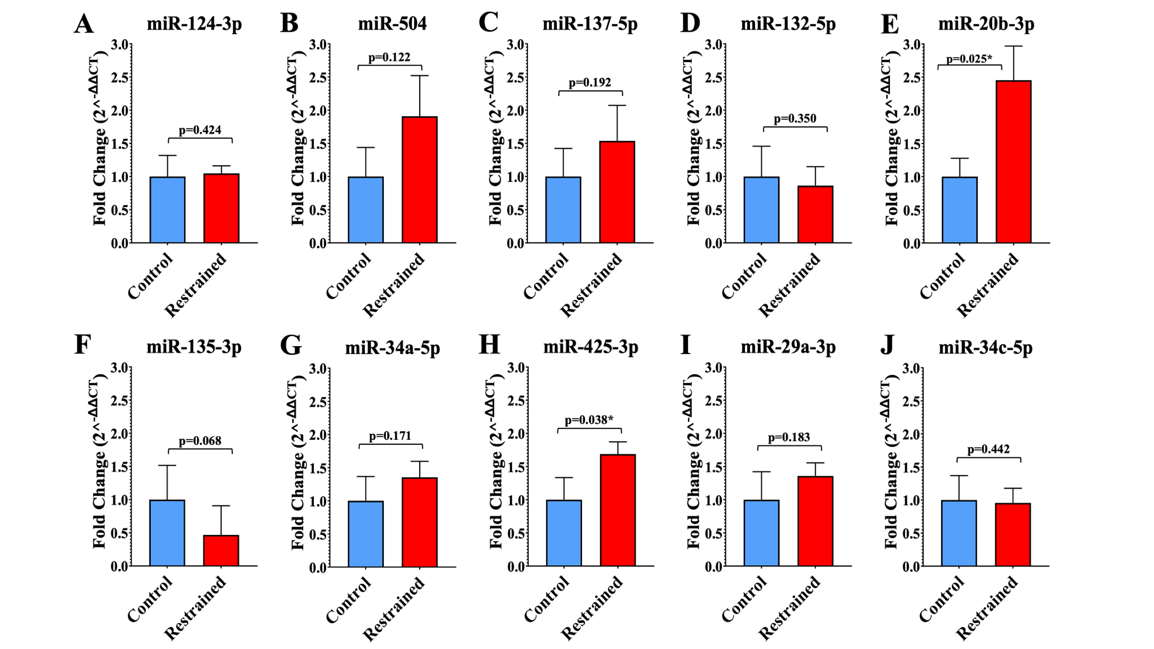


**Figure 2: Fold change analysis of stress-related miRNAs in the PFC of handled-control and restraint-stressed rats**. The relative miRNA transcript levels were analyzed using independent samples t-test in SPSS statistical software. Data are presented as mean ± SEM. 6 control rats and 6 restraint rats were analyzed. **(A)** For miR-124-3p, no substantial change was observed between restraint animals and handled-control rats (p=0.424, F=6.399, t=0.198, df=10). **(B)** miR-504 showed increased expression in the restraint group, though this change was not statistically significant (p=0.122, F=0.236, t=1.240, df=10). **(C)** Similarly, miR-137-5p expression was elevated in restraint-stressed rats but remained above the significance threshold (p=0.192, F=0.347, t=0.909, df=10). **(D)** miR-132-5p displayed a slight decrease in the restraint group without statistical significance (p=0.350, F=3.594, t=-0.396, df=10). **(E)** In contrast, miR-20b-3p was significantly upregulated in restraint animals compared to controls, indicating a robust increase in expression in response to chronic stress (p=0.025, F=4.022, t=2.219, df=10). **(F)** miR-135-3p was downregulated in restraint-stressed rats, with a trend toward significance that did not meet the p ≤ 0.05 threshold (p=0.068, F=0.027, t=1.620, df=10). **(G)** miR-34a-5p exhibited an increase in the restraint group, but this change was not significant (p=0.171, F=1.953, t=0.998, df=10). **(H)** At a statistically significant level, miR-425-3p was elevated in restraint animals compared to controls (p=0.038, F=2.861, t=1.984, df=10). **(I)** miR-29a-3p expression increased following restraint stress, but did not reach significance (p=0.183, F=6.600, t=0.948, df=10). **(J)** miR-34c-5p expression remained stable across both groups, with no notable differences observed (p=0.442, F=1.288, t=-0.149, df=10). All miRNA expression data was normalized against U6. *p ≤ 0.05.


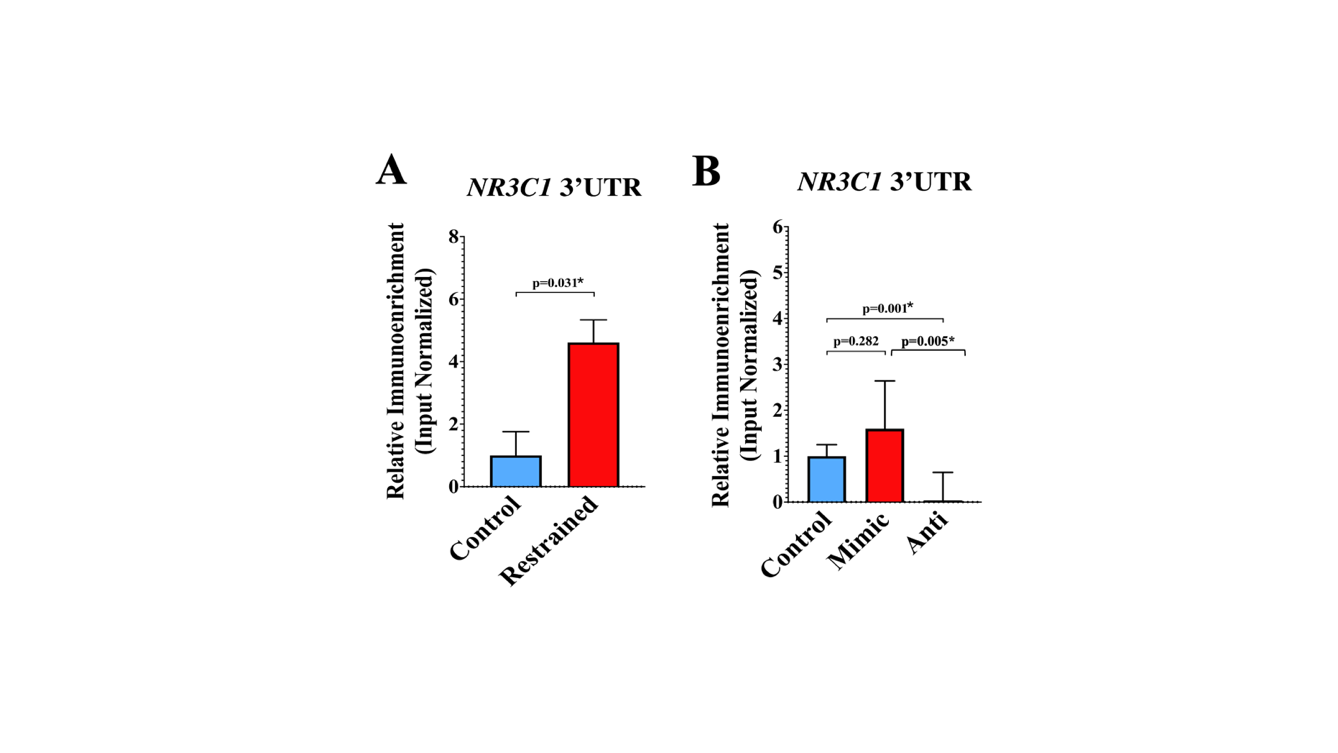


**Figure 3: RISC-mediated fold change differences of *NR3C1* 3′UTR by miR-20b-3p in restraint-stressed rat PFC and miR-20b-3p oligo-transfected cells. (A)** In vivo analysis of PFC tissue from restraint-stressed (n=5) and handled-control rats (n=5) revealed a significant increase in *NR3C1* 3′UTR enrichment in the restraint group (p=0.035, F=0.029, t=2.086, df=8), suggesting enhanced miR-20b-3p-mediated targeting of *NR3C1* transcripts in response to chronic stress. **(B)** In vitro, PC-12 Adh cells were transfected with a miR-20b-3p agomir (mimic, n=3) or inhibitor (anti-miRNA, n=3) and compared to a non-transfected control group (n=3). Although the increase did not reach statistical significance (p=0.282), ectopic overexpression of miR-20b-3p was associated with enhanced binding to the *NR3C1* 3′UTR. In contrast, this interaction was markedly reduced in cells transfected with the miR-20b-3p inhibitor (p=0.001), supporting the role of miR-20b-3p as a direct post-transcriptional regulator of *NR3C1*. All statistical analyses were performed using Student’s t-test and independent samples t-test in SPSS statistical software. Data are presented as mean ± SEM. *p ≤ 0.05. Ago2 = Argonaute 2; RIP = RNA immunoprecipitation; 3’UTR = 3’ untranslated region.
